# Radiomics Based on CECT in Differentiating Kimura Disease From Lymph Node Metastases in Head and Neck: A Non-Invasive and Reliable Method

**DOI:** 10.3389/fonc.2020.01121

**Published:** 2020-07-27

**Authors:** Ying Zhang, Shujing Yu, Li Zhang, Liqing Kang

**Affiliations:** ^1^Graduate School, Tianjin Medical University, Tianjin, China; ^2^Department of CT Diagnosis, Cangzhou Central Hospital, Cangzhou, China; ^3^Department of Magnetic Resonance Imaging, Cangzhou Central Hospital, Cangzhou, China

**Keywords:** Kimura disease, lymph node, metastases, radiomics, nomogram, texture analysis, differential diagnosis, CT

## Abstract

**Background:** Kimura disease may be easily misdiagnosed as malignant tumors such as lymph node metastases based on imaging and clinical symptoms. The aim of this article is to investigate whether the radiomic features and the model based on the features on venous-phase contrast-enhanced CT (CECT) images can distinguish Kimura disease from lymph node metastases in the head and neck.

**Methods:** A retrospective analysis of 14 patients of head and neck Kimura disease (a total of 38 enlarged lymph nodes) and 39 patients with head and neck lymph node metastases (a total of 39 enlarged lymph nodes), confirmed by biopsy or surgery resection, was conducted. All patients accepted CECT within 10 days before biopsy or surgery resection. Radiomic features based on venous-phase CECT were generated automatically from Artificial-Intelligence Kit (AK) software. All lymph nodes were randomly divided into the training set (*n* = 54) and testing set (*n* = 23) in a ratio of 7:3. ANOVA + Mann–Whitney, Spearman correlation, least absolute shrinkage and selection operator, and Gradient Descent were introduced for the reduction of the highly redundant features. Binary logistic regression model was constructed based on the selected features. Receiver operating characteristic was used to evaluate the diagnostic performance of the features and the model. Finally, a nomogram was established for model application.

**Results**: Seven features were screened out at the end. Significant difference was found between the two groups for all the features with area under the curves (AUCs) ranging from 0.759 to 0.915. The AUC of the model's identification performance was 0.970 in the training group and 0.977 in the testing group. The disease discrimination efficiency of the model was better than that of any single feature.

**Conclusions**: The radiomic features and the model based on these features on venous-phase CECT images had very good performance for the discrimination between Kimura disease and lymph node metastases in the head and neck.

## Introduction

Kimura disease, also known as eosinophilic lymphogranuloma, is a rare lymphoproliferative disease, which occurs in the head and neck with unknown origin and expounded systemically in 1948 by Kimura (1). The clinical symptoms of Kimura disease include a painless soft tissue mass, with peripheral lymphadenopathy or lymphadenectasis in the neck and submandibular region (2). The imaging findings of Kimura disease are non-specific; even though the lesions may have some features such as well-defined boundaries, lack of liquefaction necrosis, and calcification or fusion trend, it is still difficult to be distinguished from other lymphadenectasis for some cancer patients with lymph node metastases without symptoms of primary tumors (3–5). The main treatment of Kimura disease is radiotherapy instead of radical surgery, which is preferred for some kind of lymph node metastases in the head and neck. So, it is essential to make an accurate differential diagnosis for clinical intervention. Currently, the diagnosis of Kimura disease is mainly based on the judgment of clinical features and the histopathological examination. However, the clinical judgment is inaccurate, and it is uneconomical and uncomfortable for patients to undergo non-comprehensive sampling and time-consuming and invasive surgical resection or biopsy. An accurate, non-invasive, and efficient method of disease identification is urgently needed.

Radiomics is a newly emerging form of imaging analysis using a series of data mining algorithms or statistical analysis tools on high-throughput imaging features extracted from radiographic data to obtain diagnostic or prognostic information (6–8). By building appropriate models with refined features, successful assessment, and prediction abilities in various challenging clinical tasks can be achieved (9–13). Recent studies of radiomics have provided insights in precision medicine in oncologic practice related to tumor detection, subtype classification, lymph node metastases, survival, and therapeutic response evaluation (14). A review walking through several steps necessary for radiomic analysis in brain tumor in detail showed how it is able to use radiomics in diseases (15). As far as we have known, the application of radiomics for differential lymph node lesions of Kimura disease from lymph node metastases in the head and neck has not been reported in the literature yet.

The purpose of this study was to investigate whether radiomic features extracted from contrast-enhanced CT (CECT) images and the model build on the features could be used for differentiating Kimura disease from metastases.

## Materials and Methods

### General Information

This retrospective study was approved by the ethics committee of Cangzhou Central Hospital, and the informed consent requirement was waived. The research method was in accordance with the standard guidelines and regulations. The clinical histopathologic and radiological data were collected from July 2011 to August 2018. The cohort inclusion criteria were as follows: (a) lymph nodes with histopathologically confirmed Kimura disease and lymph node metastases in head and neck by means of biopsy or surgery resection, (b) patients with CECT performed within 10 days before the pathological examination, and (c) lymph nodes without liquefaction necrosis or calcification with the minimum diameter not <1.0 cm (4, 16). The exclusion criteria were (a) poor image quality with artifacts and (b) patients who had previously received related therapy. Eventually, a total of 77 lymph nodes were included in our study—among them were 14 patients (12 males and two females; mean age, 36.5 years old; range, 16–51 years) with a total of 38 lymph nodes diagnosed as Kimura disease in head and neck. Eight lymph nodes were located in level I, 10 in level II, one in level III, two in level V, and 17 in level VIII. There were 39 patients (20 males and 19 females; mean age, 59.2 years old; range, 30–77 years) with 39 lesions diagnosed as lymph node metastases. Ten lymph nodes were located in level II, eight in level III, 14 in level IV, five in level V, and two in level VI. The level of lymph nodes is defined according to the method described by Gregoire et al. (17). The lymph node metastases were derived from variously sourced cancerous foci (see Table S1 for detailed information).

### CT Image Acquisition

All enrolled patients underwent CECT examination (Light Speed 64, Waukesha, WI, USA). All patients took the supine position. The range of the scan was from the skull base to the sternal notch. The scan parameters were as follows: tube of voltage of 120 KV, tube current of auto Am, slice thickness 2.5 mm, interval 2.5 mm, and pitch 1.375. Ultravist (350 mg I/Ml, 1.5 ml/kg) was injected with a rate of 3.5 ml/s through the elbow vein by a high-pressure injection. Axial arterial-phase and venous-phase CT images were obtained at 25–30 and 60–70 s after injection and were exported in DICOM format.

### Radiomic Features

#### VOI Segmentation and Radiomic Feature Acquisition

**Figure 1:**
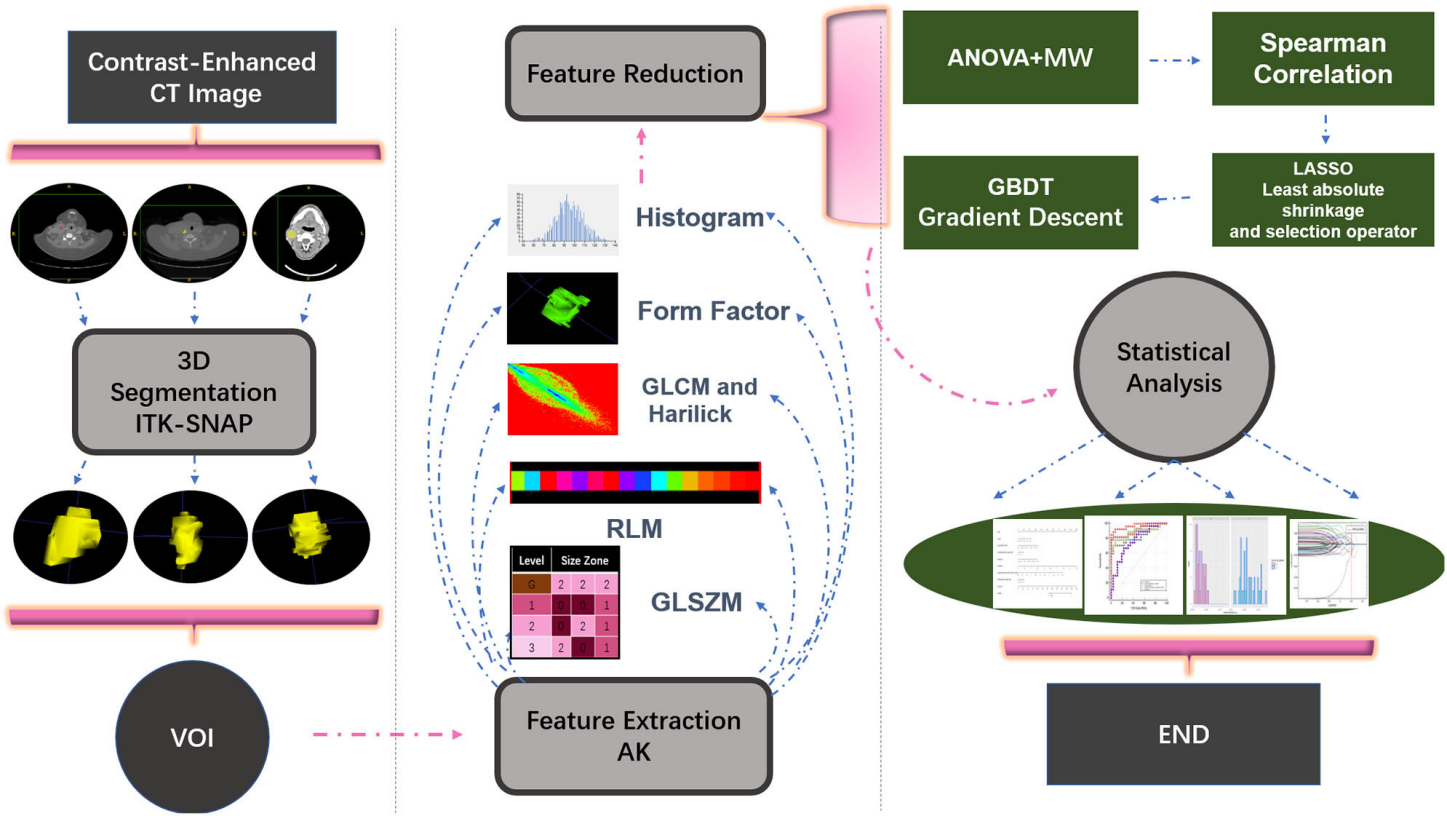
Illustration of the process of data analysis. First, each layer of the lesion was manually segmented and automatically merged into a three-dimensional volume of interest (VOI) in the software. Then, upon extraction of the VOI radiomic features, relevant statistical methods were carried out for feature dimensionality reduction, and finally a statistical analysis on the selected features was performed and a model classifier was established.

**Figure 2:**
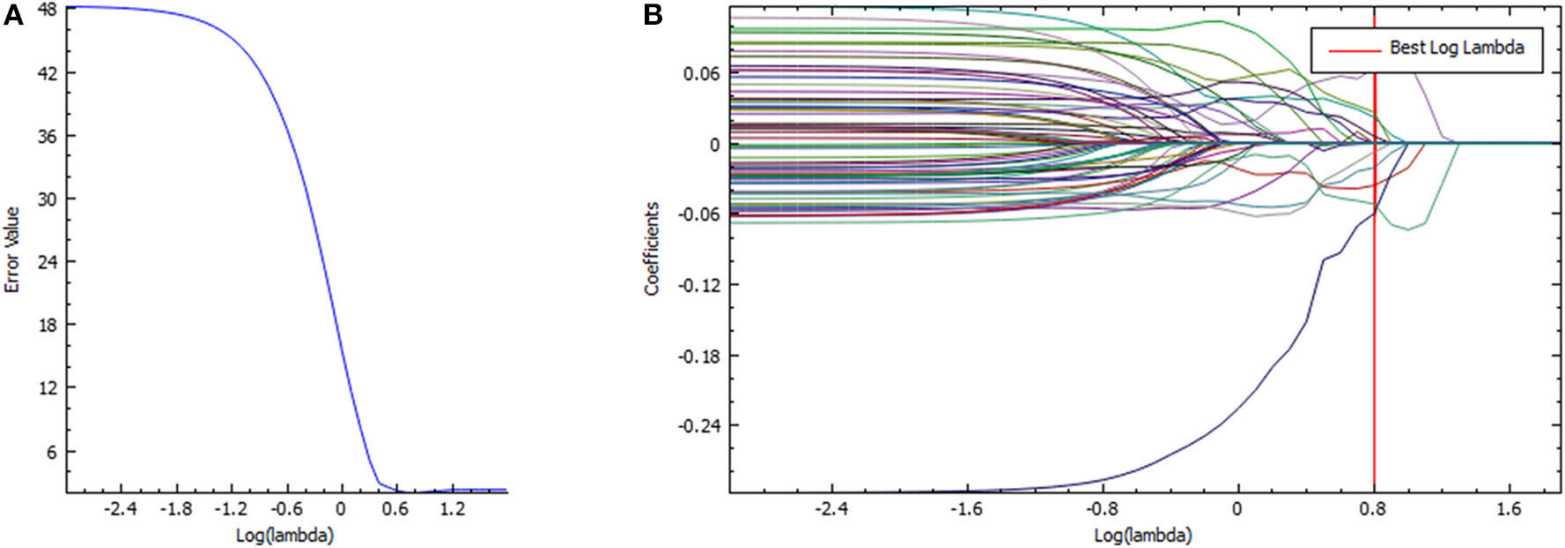
Feature selection in the LASSO model. **(A)** LASSO error graph tuning parameter (λ) selection in the LASSO model used 10-fold cross-validation *via* minimum criteria. The error value was plotted vs. log(λ). Seven features with the smallest error value were selected. **(B)** LASSO coefficient profiles of the texture features. The red vertical line is drawn at the value selected using 10-fold cross-validation in log(λ) sequence and coefficients with non-zero values are indicated.

The venous-phase images were used for image feature extraction as the distribution of the contrast agent in the lesions was more homogeneous, and the image quality was better for distinguishing the lesions from the adjacent tissue (18–20). The technical process of the entire study is shown in Figure 1. The lesions were delineated on the venous-phase CECT images using the ITK-SNAP software (available at www.itksnap.org) in soft-tissue window (window width, 35; window level, 400). Two experienced radiologists (ZY, reader #1, radiology resident; ZL, reader #2; both doctors have 10 years of experience in imaging) blinded to the clinical outcomes were involved in ROI segmentation. The whole-tumor volume was determined by manually drawing a region of interest along the border of the tumor on each consecutive slice covering the whole lesion. Therefore, a three-dimensional (3D) volume of interest (VOI) was finally obtained. The radiomic features were automatically calculated by AK software (Artificial Intelligence Kit, GE Healthcare, Shanghai, China). The features extracted by the AK software comply with the standards set by the Image Biomarker Standardization Initiative. In total, 396 imaging features were extracted in each lesion, including (1) histogram features, such as mean, uniformity, skewness, kurtosis, energy, and entropy; (2) form factor features, such as volume CC, surface, surface volume ratio, compactness, and maximum 3D diameter; and (3) texture features including Gray level co-occurrence matrix (GLCM), Gray level run length matrix (GLRLM), Gray level size zone matrix, and Haralick parameters. The offset of GLCM and GLRLM were 1, 4, and 7. Features pre-processing was conducted in two steps: step 1—outliers and null values were replaced by mean values, and step 2—values standardization was carried out to eliminate the influence of the dimension. Feature dimension reduction was performed as follows: First, analysis of variance (ANOVA) and Mann–Whitney *U*-test were performed. Second, Spearman correlation test was conducted to remove the highly correlated variables. Third, in the LASSO model, the value of the minimum error rate among the 10-fold cross-validation was selected to construct the penalty function to compress the unimportant variable coefficients to zero (Figure 2). Gradient Descent algorithm for further feature screening was performed when the features were still redundant. In the study, the morphological features of the lesions were excluded. An analysis was made only about the texture features of the lesions (21).

#### Radiomic Modeling and Validation

All lymph nodes were randomly divided into training set (*n* = 54) and testing set (*n* = 23) with a ratio of 7:3. A total of 27 of the 38 lymph nodes of Kimura disease and 27 of 39 lymph nodes of metastases were included in the training set and 11 other lymph nodes of Kimura disease and 12 lymph nodes of metastases were in the testing set. Then, the binary logistic regression model was constructed based on the training set data to validate the model with the testing set data. The features and model identification performance were quantified by the area under the receiver operating characteristic (ROC) curve (AUC) in the training and the testing sets. Radiomic nomogram was then constructed on the basis of the binary logistic regression model. Radscore was calculated for each lesion and then converted into the risking probability of lymph node metastasis. A decision curve analysis was performed to evaluate the clinical benefit of the nomogram model developed in the testing dataset. The x axis of the curve is the threshold of the predicted probability outcome by the nomogram model. The y axis is the clinical decision net benefit for patients based on the discrimination result under this threshold.

### Statistical Analysis

Statistical analysis was performed by R studio (1.1.463, packages: “verification,” “pROC,” “rms,” “glmnet,” “caret,” and “rmda”) and IBM SPSS Statistics 22. With regard to the reproducibility of volumetric and texture analysis, inter-observer reliability was assessed by intra-class correlation coefficient (ICC) test. Delong test was used for significant difference test among AUCs (22). Hosmer–Lemeshow test was used for evaluating model fit-goodness. The normal distribution test was performed using Shapiro–Wilk on continuous quantitative variables. Levene's test was used for equality of variances. *P* > 0.05 was considered to be normal distribution and variance is equal. Independent-sample *t*-test was used for significant difference in variable distribution if normally distributed; otherwise, the non-parametric Mann–Whitney *U*-test was used. The qualitative variables were compared with chi-square or Fisher's exact test. *P* < 0.05 was considered as statistically significant.

## Results

**Figure 3:**
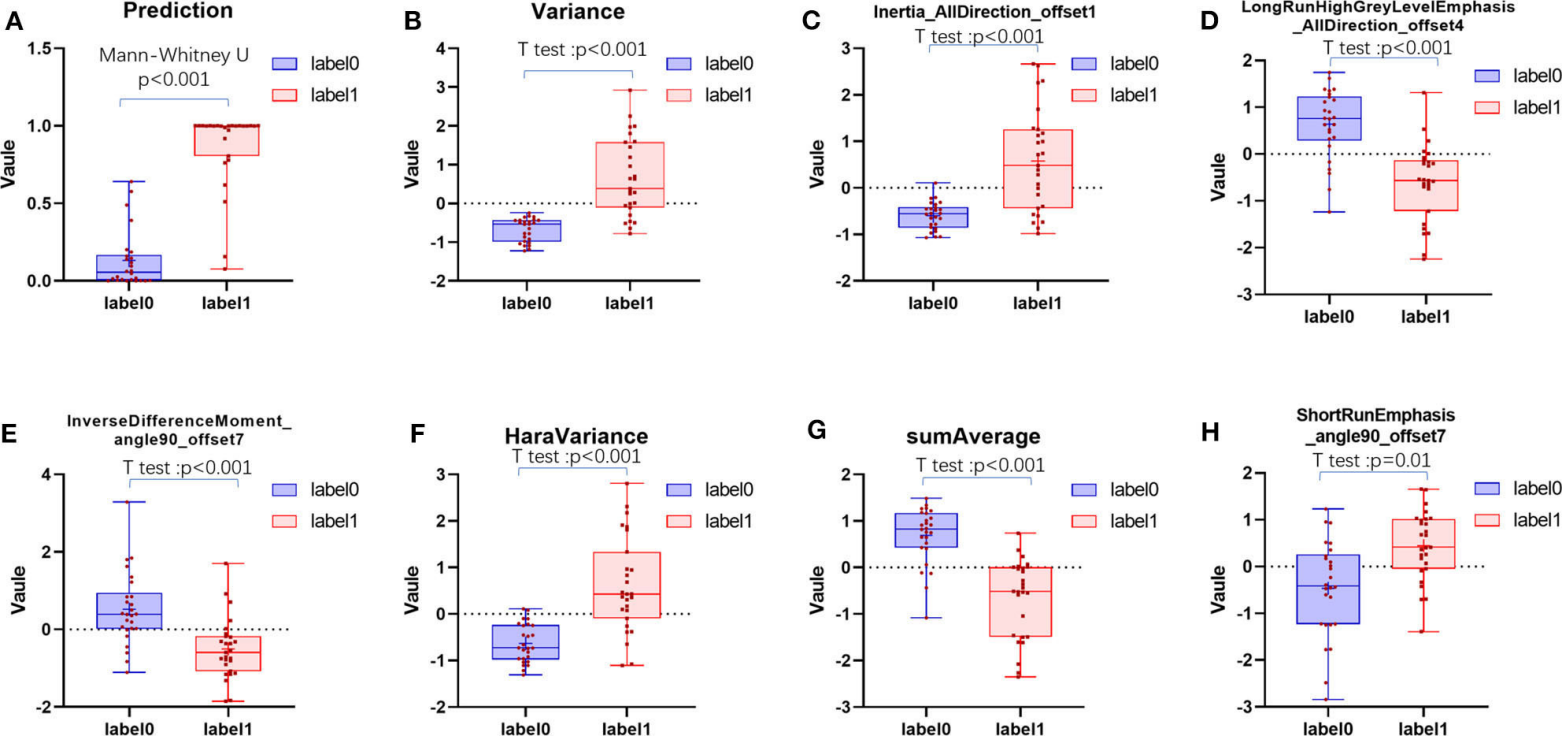
The distribution of positive prediction probability and the selected feature values between the two groups **(A–H)**. Label 0 represents the Kimura disease group and the label 1 represents the lymph node metastases group. There was a significant difference in the distribution of values between the two groups, and all *P-*values were <0.05.

**Figure 4:**
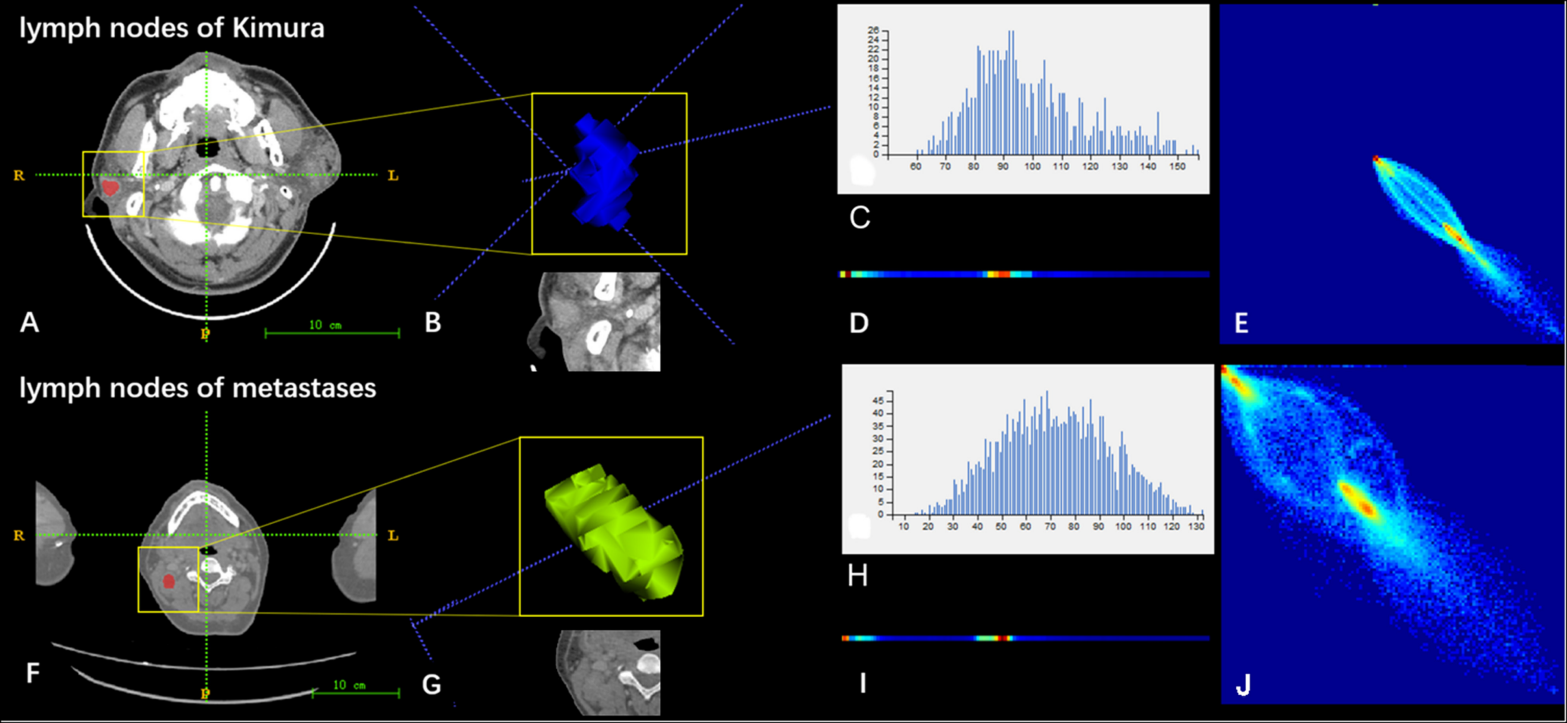
Two sets of mapped images of radiomic features of patients with Kimura disease **(A–E)** and lymph node metastases **(F–J)**. **(A,F)** CECT images. **(B,G)** Volumes of interest. **(C,H)** Grayscale distribution histogram of the lesions. The distribution of **(H)** was more dispersed than the distribution of **(C)**. **(D,I)** Run length matrix features. The frequency of changing of the gray scale of **(D)** was smaller than that of **(I)**. **(E,J)** Gray level co-occurrence matrix features. The heterogeneity of lesions in **(J)** was greater than that of **(E)**.

The ICC values of the inter-observer of our research were 0.76–0.97, which suggest great accordance between two readers and the reliability of VOI sketching (22, 23). Three hundred ninety-six radiomic features were extracted automatically by AK software. The morphological features of the lesions were excluded and seven features were left after the redundancy reduction step, including one histogram feature, four GLCM features, and two GLRLM features. The seven features were significantly different between the two groups (all *P* < 0.05) (Figure 3, Figure S1). In the histogram feature of variance, the first quantile of the lymph nodes of the metastases group was significantly higher than the maximum value of the lymph nodes of the Kimura disease group. The variance value of metastases is generally greater than that of Kimura disease, which suggests that the image-brightness-changing gradient of metastases was steeper than that of Kimura disease. In the GLCM feature cluster, the first quantile of the Inertia_AllDirection_offset1 and HaraVariance of the metastases group is slightly larger than the fourth quantile of the Kimura group. The greater the value, the greater is the difference in the lesions. While the first quantile of the Kimura group of InverseDifferenceMoment_angle90_offset7 and sumAverage was greater than the fourth quantile of the metastases group, the larger the value of these two features, the smaller is the lesion difference. In the GLRLM cluster, the first quantile of the LongRunHighGreyLevelEmphasis_AllDirection_offset4 feature of the Kimura group was significantly higher than the fourth quantile of the metastases group, while in the ShortRunEmphasis_angle90_offset7 feature, the median of the metastases group was greater than the fourth quantile of the Kimura group. The first quantile of the metastases group is slightly lower than that of the Kimura group. The larger the value, the greater is the difference in gray value between adjacent pixels in the lesion (Figure 3). Two sets of mapped images of CECT and radiomic features of patients with Kimura disease and lymph node metastases are shown, respectively, in Figure 4, wherein the histogram is the gray scale distribution of the entire lesion. The gray distribution of the Kimura disease patient is more concentrated than that of the metastases patient. The variation of the run length matrix of metastases patients is greater than that of the Kimura patients, and the GLCM shows that the lesion complexity of the Kimura disease patient is less than that of the metastases patient.

### Radiomic Model Building and Validation

A binary logistic regression model was established using the seven distinctive features. The radscore value of each lesion (Formula 1) was obtained, and the predicting risking probability of lymph nodes of metastases was obtained (Formula 2).

(1) $$\begin{array}{r} Radscore=4.290+8.476A+2.587B-2.232C\\ +\;7.690D+1.142E+0.092F-10.934G \end{array}$$

(2) $$\begin{array}{r} Probability\;positive\;prediction\;probability=\frac{1}{1+e^{\left(-radscore\right)}} \end{array}$$

(A: Variance, B: Inertia_AllDirection_offset1, C: HaraVariance, D: LongRunHighGreyLevelEmphasis_AllDirection_offset4, E: ShortRunEmphasis_angle90_offset7, F: InverseDifferenceMoment_angle90_offset7, and G: sumAverage).

If the coefficient of the variable is negative, the smaller the value, the greater the risk probability will be. If the coefficient of the variable is positive, the smaller the value, the smaller the risk of developing lymph nodes of metastases will be.

**Figure 5:**
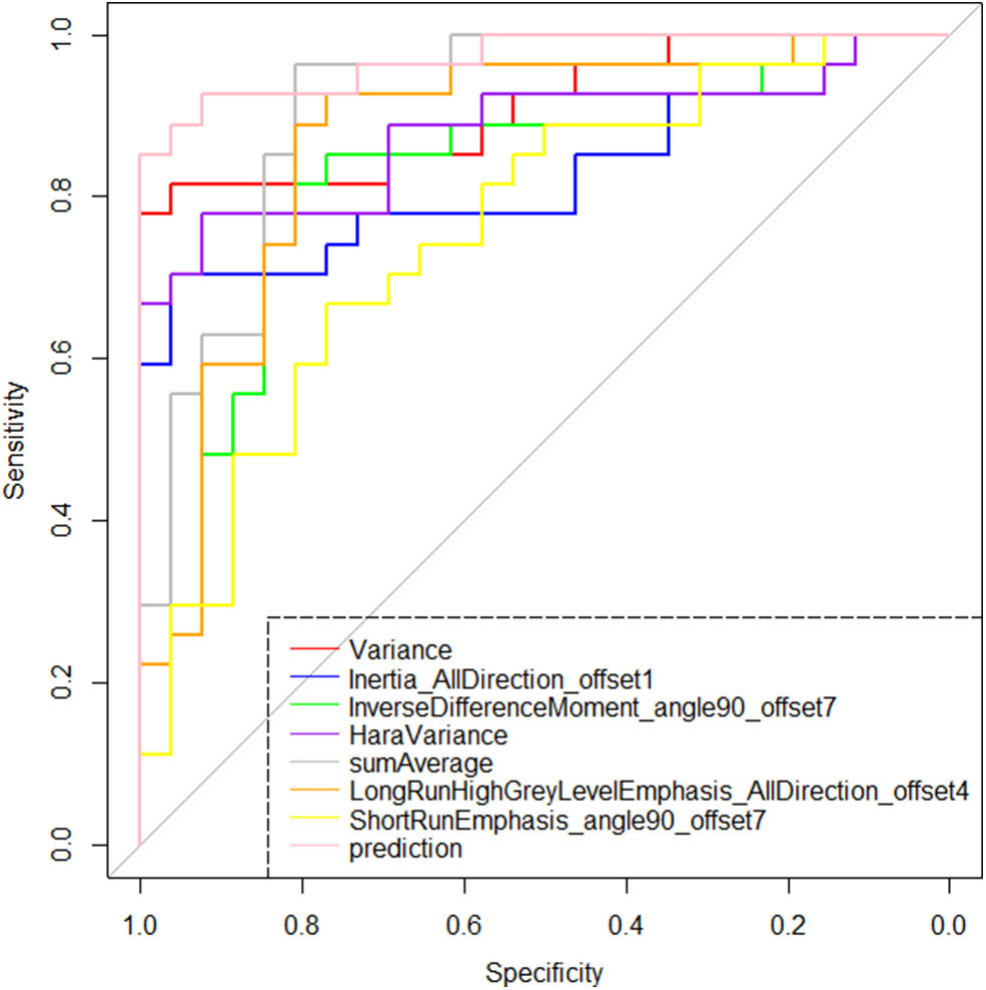
Receiver operating characteristic plots of the selected seven features and the model's prediction probability based on them. According to the area under the curve index, the identification effectiveness of the binary logistic regression model based on the seven features is greater than the discrimination performance of any single feature.

The radiomic signature showed favorable predictive efficacy. The risking probability according to radscore shows a significant difference between the two groups (*P* < 0.001) (Figure 3). As can be seen from the figure, the prediction probability of the lymph nodes of the Kimura disease group is much lower than that of the lymph nodes of the metastases group [cutoff value: 0.490—this cutoff value is taking into account disease prevalence (50.9%); the value larger than 0.490 is considered to be the metastasis group, while the value smaller than 0.490 is thought to be the Kimura disease group]. The positive and negative predictive values of prediction were higher than the performance values of the other seven features. According to the OR values of the seven features, the importance of the features can be ranked, where extreme values <1 or >1 indicate that the feature is more important, and the values of sumAverage and variance are more extreme, so the contribution of these two features to the model was greater, which is consistent with their AUC value trend (Table 1). The ROC curves of seven features and the predicted probabilities are established to evaluate the performance of each feature and model (Figure 5). From the figure, we can see that the model prediction probability (AUC: 0.970) is optimal for disease detection, followed by sumAverage (AUC: 0.915) and variance (AUC: 0.910). The AUC value of each variable was significantly different from the AUC of 0.5 (all *P* < 0.0001), indicating that each variable is reliable for the prediction and the identification of the disease. According to the AUC DeLong test between prediction and the other seven features, except for sumAverage and variance, the predictive power of prediction was significantly different from the other five features (all *P* < 0.05). All the information above can be seen in Table 2. In general, the eight variables in the figure have a good distinguishing effect on the disease. Sensitivity and specificity are obtained according to the most approximate Youden index. The optimal criterion value indicates the cutoff value which was assigned to the metastatic tumor group.

**Table 1:** The parameters of the model and the seven features of disease identification performance.

**Features**	**AUC (95% CI)**	**Sensitivity**	**Specificity**	**Optimal criterion**	**Significance level *P* (area = 0.5)**	**OR**	**+LR (95% CI)**	**-LR (95% CI)**	**+PV (95% CI)**	**-PV (95% CI)**	**Cost**
Variance	0.910 (0.830–0.991)	81.48	96.15	>-0.329	<0.0001	4800.048	21.19 (3.1–146.0)	0.19 (0.09–0.4)	95.7 (76.1–99.3)	83.3 (69.3–91.7)	0.113
Inertia_AllDirection_offset1	0.828 (0.711–0.945)	70.37	96.15	>-0.205	<0.0001	13.296	18.30 (2.6-127.0)	0.31 (0.2-0.6)	95.0 (73.2-99.2)	75.8 (63.5-84.9)	0.170
InverseDifferenceMoment_angle90_offset7	0.823 (0.705–0.942)	77.78	84.62	≤ -0.179	<0.0001	1.096	5.06 (2.0-12.7)	0.26 (0.1–0.5)	84 (67.6–93.0)	78.6 (64.0–88.3)	0.189
HaraVariance	0.879 (0.779–0.978)	77.78	92.31	>-0.098	<0.0001	0.015	10.11 (2.6–38.9)	0.24 (0.1–0.5)	91.3 (73.2–97.6)	80 (66.2–89.1)	0.151
sumAverage	0.915 (0.837–0.99)	96.30	80.77	≤ 0.373	<0.0001	0	5.01 (2.3–11.0)	0.046 (0.007–0.3)	83.9 (70.2–92.0)	95.5 (75.3–99.3)	0.113
LongRunHighGreyLevelEmphasis_AllDirection_offset4	0.869 (0.766–0.972)	92.59	76.92	≤ 0.281	<0.0001	2,187.088	4.01 (2.0–8.2)	0.096 (0.02–0.4)	80.6 (67.2–89.4)	90.9 (72.2–97.5)	0.151
ShortRunEmphasis_angle90_offset7	0.759 (0.629–0.890)	66.67	76.92	>0.228	<0.0001	3.133	2.89 (1.4–6.1)	0.43 (0.2–0.8)	75 (58.6–86.4)	69 (55.6–79.8)	0.283
Prediction	0.970 (0.931–1)	92.59	92.31	>0.490	<0.0001	-	12.04 (3.2–45.8)	0.080 (0.02–0.3)	92.6 (76.7–97.9)	92.3 (75.9–97.9)	0.076

*+LR, positive likelihood ratio; -LR, negative likelihood ratio; +PV, positive predictive value; -PV, negative predictive value; 95% CI, 95% confidence interval (binomial exact)*.

**Table 2:** Significant difference test between area under the curve values of the model and the seven features.

**Comparison of variables**	**Difference between areas**	**Standard error**	**95% confidence interval**	***z* statistic**	**Significance level**
Prediction ~ HaraVariance	0.0912	0.0417	0.00945 to 0.173	2.187	*P* = 0.0288
Prediction ~ LongRunHighGreyLevelEmphasis_AllDirection_offset4	0.101	0.05	0.00306 to 0.199	2.021	*P* = 0.0433
Prediction ~ Inertia_AllDirection_offset1	0.142	0.0521	0.0403 to 0.245	2.733	*P* = 0.0063
Prediction ~ ShortRunEmphasis_angle90_offset7	0.211	0.0623	0.0887 to 0.333	3.384	*P* = 0.0007
Prediction ~ InverseDifferenceMoment_angle90_offset7	0.147	0.0563	0.0363 to 0.257	2.604	*P* = 0.0092
Prediction ~ sumAverage	0.0556	0.0361	−0.0152 to 0.126	1.54	*P* = 0.1236
Prediction ~ Variance	0.0598	0.0313	−0.00152 to 0.121	1.911	*P* = 0.0560

**Figure 6:**
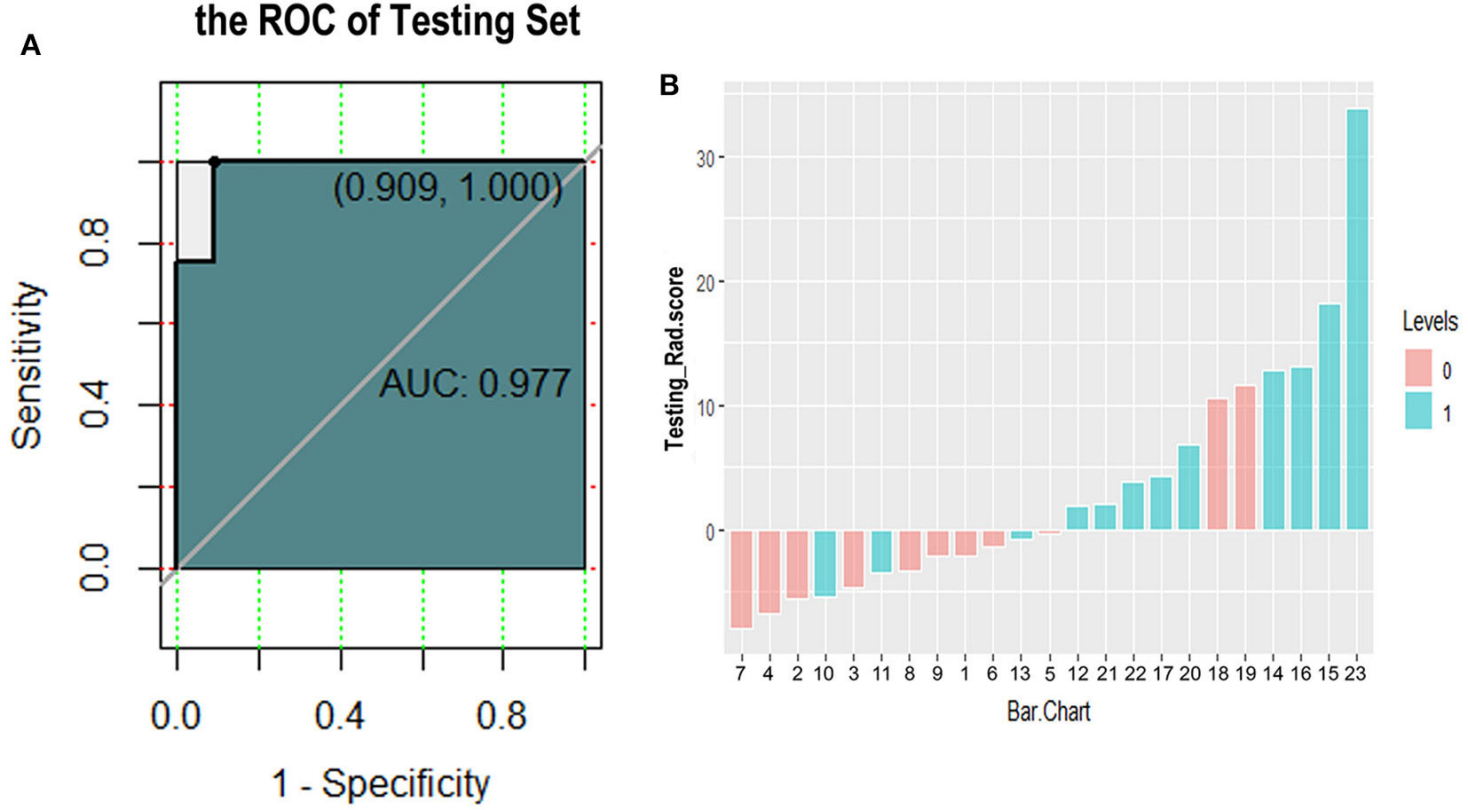
Receiver operating characteristic curve **(A)** and classification effect diagram **(B)** for verifying the model with testing data. **(A)** The area under the curve is 0.977, the sensitivity is 1.0, and the specificity is 0.909 in testing data. **(B)** Pink and blue represent label 0 and label 1 according to the gold standard, respectively. The model uses 0 as the classification threshold, with blues <0 and pinks >0 being cases of model misclassification. It can be seen that the classification effect of the model in the testing group is generally good.

**Figure 7:**
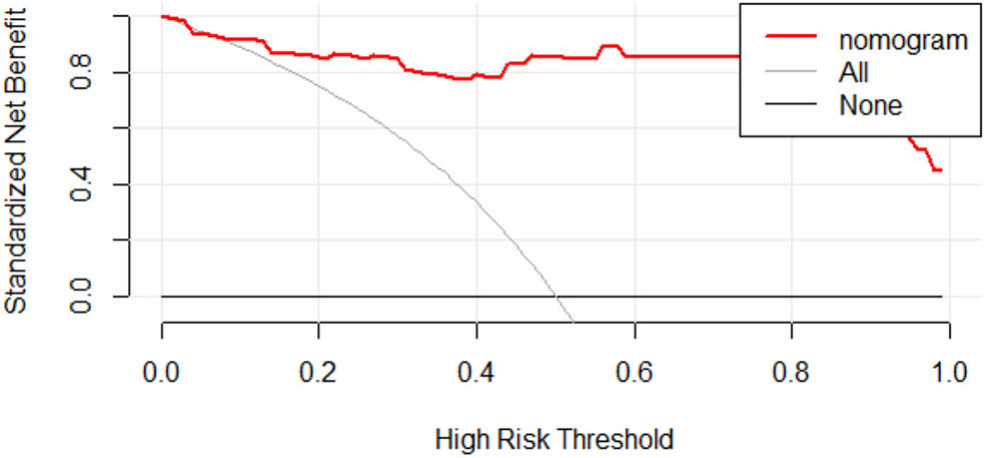
Decision curve analysis for radiomic discrimination model. The Y-axis represents the standardized net benefit. All: assuming that all patients will be treated. None: assuming that no patient will be treated. Red line: the nomogram prediction performance based on model. When making a decision based on a nomogram, the standard net benefit obtained is greater than the treatment of all patients or none in the range of threshold probability 0.08–1.0.

We further tested the fit-goodness of the established binary logistic regression model. The results show that the model is in good agreement with the actual model (χ^2^ = 2.127, *P* = 0.977, Hosmer–Lemeshow test). The model was validated in the testing group and found to have good generalization ability. The AUC of the testing set was 0.977, the sensitivity was 1, and the specificity was 0.909 (Figure 6A). The model performed to be a good classifier on the testing set data (Figure 6B). The decision curve of the model shows that the net benefit of making decisions based on the established model is much greater than treating all patients or not treating all patients between the probability threshold of 0.08–1.0 (Figure 7).

**Figure 8:**
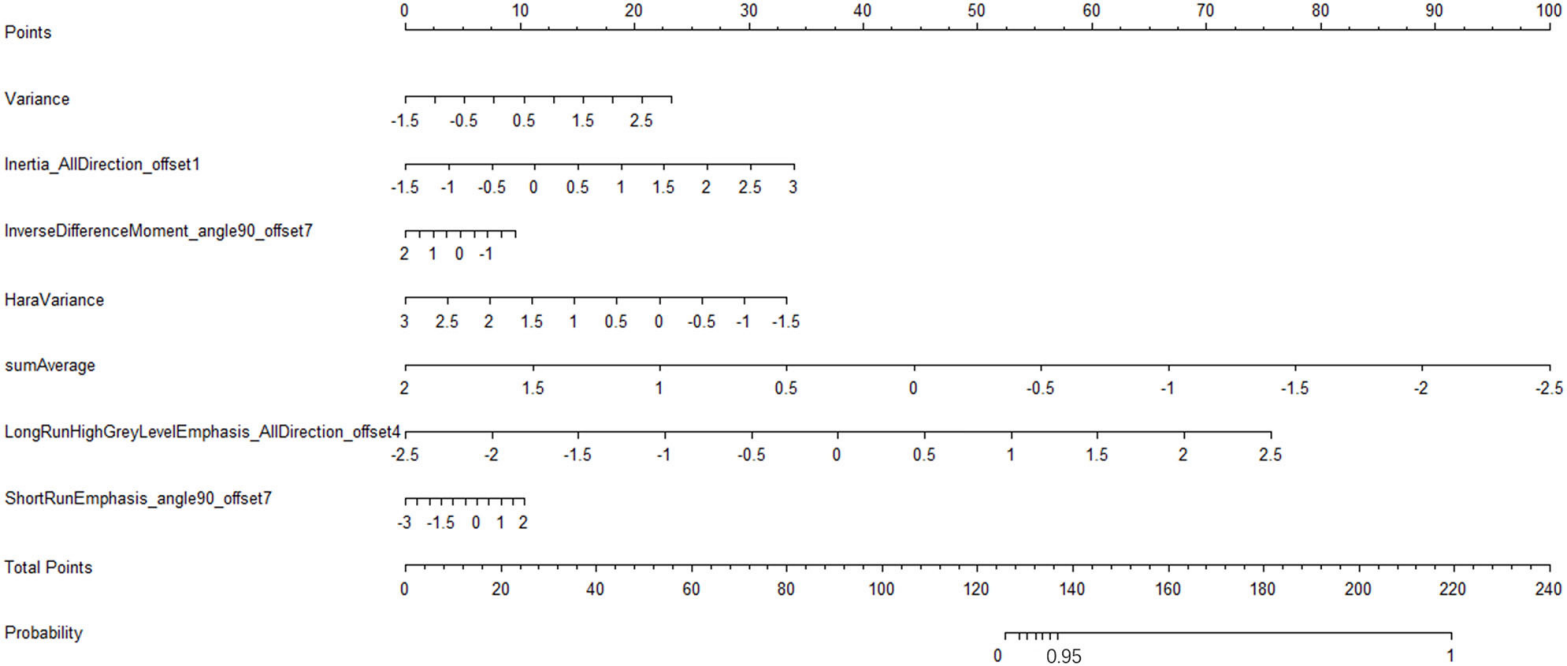
Radiomic nomogram to detect lymph node lesion. The radiomic nomogram was developed in the training set with the seven features. In the nomogram, first, make a vertical line according to the value of the selected seven features to determine the corresponding value of points. Then, the total points are the sum of the seven points above. Finally, make a vertical line according to the value of the total points to determine the probability of lymph node metastases.

We have constructed a nomogram of the predictive model for model application. After we get the patient's image feature data, normalize the feature, then get the corresponding points according to the values of these seven features, and finally add these seven points to get the total point, the total point is vertically corresponding to the probability scale line. The probability of having a metastatic tumor in this patient is available (Figure 8).

## Discussion

The present study is the first to distinguish Kimura disease from lymph node metastases in the head and neck using radiomic features and the model based on the features on venous-phase CECT images. In our study, seven features and predicted probabilities have a good distinguishing effect on the disease. The predicted probabilities are optimal for disease detection.

The morbidity of Kimura disease is low, and the majority of reports about Kimura disease focus on clinical findings, with a few of the imaging findings reported (24–26). There are three types of CT manifestations of Kimura disease in the head and neck: (1) multiple nodular type, showing multiple nodules, with clear borders and uniform enhancement, (2) diffuse mass type, characterized by ill-defined diffuse subcutaneous soft tissue mass, with thickening of the adjacent skin and mild to moderate heterogeneous enhancement, mainly located in the subcutaneous fat space of the maxillofacial region, and (3) mixed type, with characteristics of both of the above types.

Most patients having subcutaneous tumor-like nodules with large parotid gland and local lymph node involvement are easy to be misdiagnosed as malignant tumors, which make a differential diagnosis difficult even using CT and MRI. Lymph node metastasis is a high-risk factor for the prognosis (27); more than one metastatic lymph node will increase the risk of recurrence (28). Metastasis of tumors to sentinel lymph nodes can predict disease progression and often guides a treatment scheme (29). In clinical practice, many patients are required for further CT or MRI to detect whether primary tumors exist. There are different treatment modalities for Kimura disease, and the postoperative recurrence rate is high (2, 26). The main treatment of Kimura disease is different from malignant tumors. There is no need to do radical surgery. Therefore, it is necessary to make a clear diagnosis before surgery. Although some scholars have summarized some imaging features of Kimura disease in the head and neck, it is necessary to combine clinical and laboratory examinations to improve the diagnostic accuracy for lacking image characteristics and the pretty low diagnostic accuracy.

In the recent years, radiomics increasingly draws attention and has demonstrated that it may be a tool that can obtain high-fidelity target information to comprehensively evaluate lesions, especially the texture features in the image that are not recognized by the naked eye and reveal the inherent heterogeneity of the tissue, reflecting the subtle differences between different tissues. Radiomics can be combined with the imaging appearance to further improve the differential diagnosis ability of the lesion (30, 31). The AK, an imaging analytic software used in this study, has been used in many research reports (32, 33). A previous study showed that radiomic feature-based CT imaging signatures allow the prediction of lymph node metastasis in cancer and could facilitate the preoperative individualized prediction of lymph node status (20).

Kimura disease is a rare disease; the lymph nodes involved in the case are often multiple. Therefore, the diseased lymph nodes were selected as the research object, and the AK software was used for feature extraction and dimensionality reduction. A total of 396 features were extracted and seven texture features were selected to identify Kimura disease from lymph node metastases, and a logistic regression model was established. In order to avoid model over-fitting, we adopted 10-fold cross-validation using training set data and testing set data for the established model. The mean AUCs of models in the two sets were 0.7812 and 0.7628. The AUC of the testing set was 0.977, which is a strong validation of the good performance index of the logistic regression model established in the study. In this study, the seven screened out features showed a significantly different distribution between the two groups, and from the point of view of the features themselves, the results showed that the heterogeneity of metastatic tumors was greater than that of Kimura disease on CECT. This conclusion may be explained by a previous basic study. Lee et al. (29), by using comparative transcriptomics and metabolomics analyses of primary and lymph node metastasis tumors in mice, found that lymph node metastasis requires that tumor cells undergo a metabolic shift toward fatty acid oxidation (FAO). Transcriptional coactivator yes-associated protein (YAP) is selectively activated in lymph node metastatic tumors, leading to the up-regulation of genes in the FAO signaling pathway. Several bioactive bile acids accumulated to high levels in metastatic lymph node metastasis, and these bile acids activated YAP in tumor cells, likely through the nuclear vitamin D receptor. The study showed that lymph node metastases are complex.

It is also obvious that both the CECT image and the radiomic features image of the lymph nodes in a metastatic tumor patient have a greater changing rate and more complexity than that of the Kimura disease (Figure 8). The discriminated efficiency of the model is better than any single feature for the two diseases, and the disease identification ability of the model, in addition to variance and sumAverage, is significantly different from the other five features. The results show that the model has a higher identification accuracy; the decision curve of the model shows a greater standard net benefit within a wide threshold probability (0.08–1.0) than treating all patients or treating no patient. So, we prefer using this model as a basis for decision making to identify these two kinds of diseases. The nomogram is one of the important applications of the model. Through the nomogram, the risk of each patient can be predicted (20, 34, 35). Using the model to classify the data of the testing set, it is found that the correctness of the classification is good, which may be due to the small amount of sample data in the testing set. This study demonstrates that radiomics can help identify Kimura disease in the head and neck and lymph node metastases, and the established nomogram can predict the risk of lymph node metastases in patients. Radiomics can be used as an intelligent-aided tech to diagnose diseases.

There are some limitations in our study. First, this is a single-institution retrospective analysis. The sample size is rather small because of the low morbidity of Kimura disease. Second, because of lack of data, we did not integrate clinical features and genetic and immunohistochemical data into a statistically predictive model. Third, this study lacks an external validation. Therefore, the sample should be expanded and multi-center independent samples are needed to further improve the accuracy of the model. In the future, some clinical data will be integrated into a statistically predictive model.

In summary, our results showed that CECT images contain much useful information which could be used to differentiate Kimura disease from lymph node metastases, but which could not be seen through naked eyes. Radiomic technology can deeply explore the image heterogeneity information, which may be an effective and non-invasive way for differential diagnosis between Kimura disease and lymph node metastases.

## Data Availability Statement

All datasets generated for this study are included in the article/Supplementary Material.

## Ethics Statement

This retrospective study was approved by the ethics committee of Cangzhou Central Hospital, and the informed consent requirement was waived.

## Author Contributions

LK is the guarantor of the article. LK, YZ, SY, and LZ contributed to the conception and design. YZ and LZ collected and assembled the data. YZ contributed to data analysis and interpretation. All the authors contributed to manuscript writing and gave final approval of the manuscript.

## Conflict of Interest

The authors declare that the research was conducted in the absence of any commercial or financial relationships that could be construed as a potential conflict of interest.

## Supplementary Material

Figure S1 Histogram distribution of the screened out seven radiomic features.

Table S1 Pathology of 39 Lymph Node Metastases.
