# Supplementary figures and images for: Radiomics Based on CECT in Differentiating Kimura Disease From Lymph Node Metastases in Head and Neck: A Non-Invasive and Reliable Method
Source: Front Oncol. 2020 Jul 27;10:1121. doi: 10.3389/fonc.2020.01121 (PMC7397819; doi:10.3389/fonc.2020.01121)

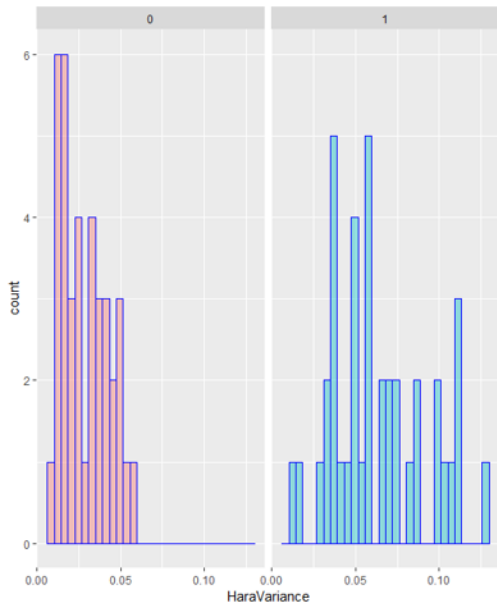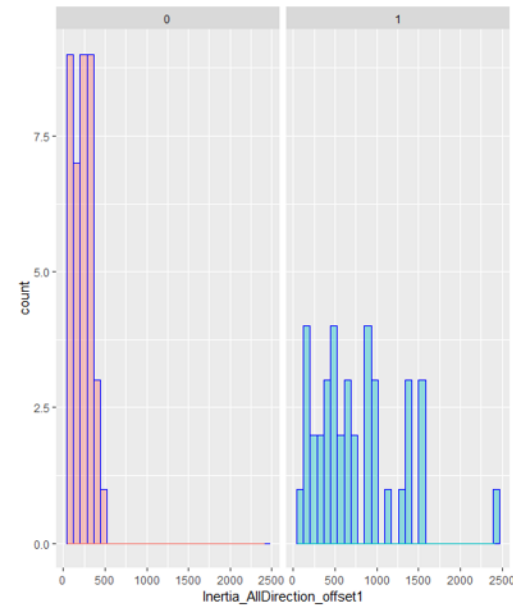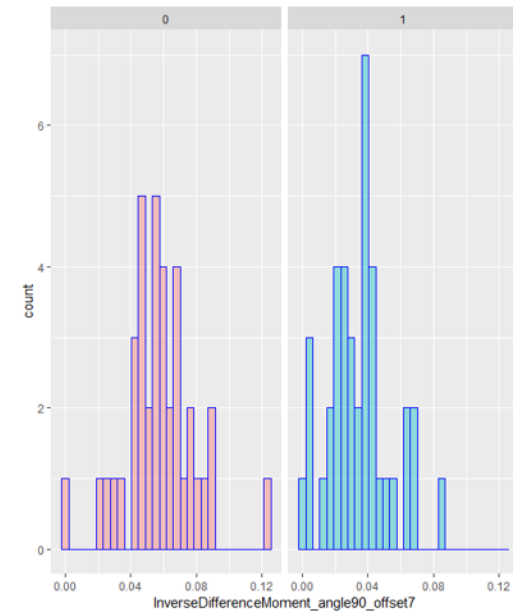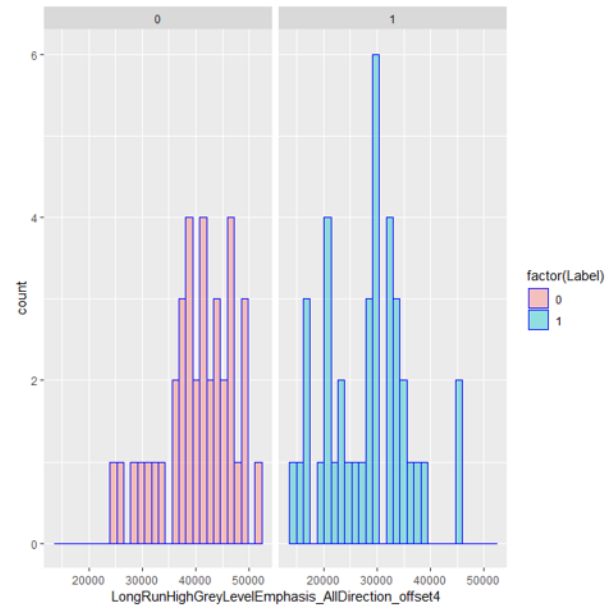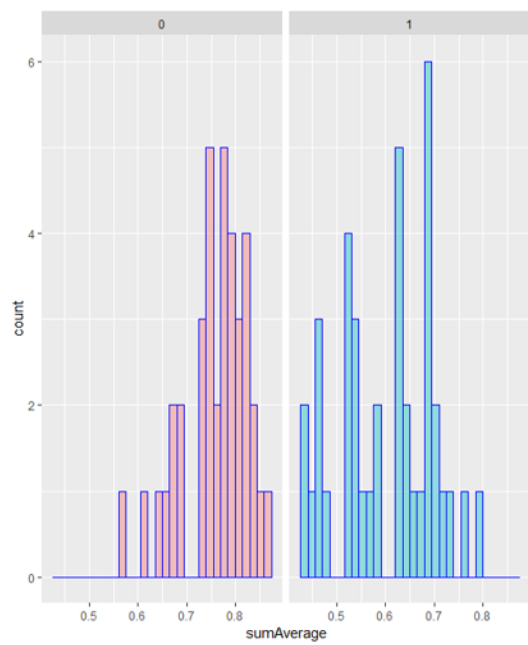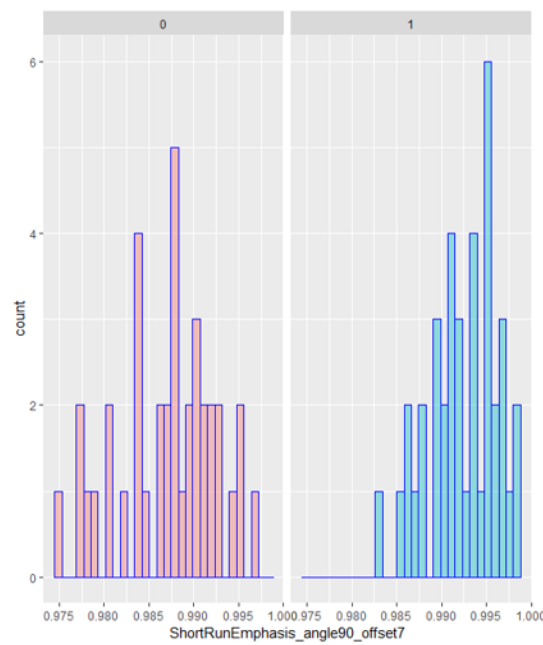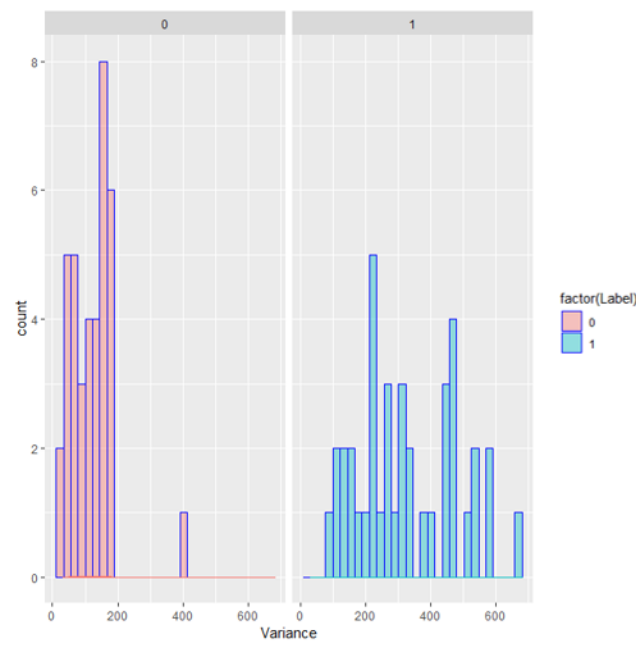

Supplement: Figure S1 — Histogram distribution of the screened out seven radiomic features. [file Image_1.pdf]
