# Supplementary material for: Radiomics Based on CECT in Differentiating Kimura Disease From Lymph Node Metastases in Head and Neck: A Non-Invasive and Reliable Method
Source: Front Oncol. 2020 Jul 27;10:1121. doi: 10.3389/fonc.2020.01121 (PMC7397819; doi:10.3389/fonc.2020.01121)
Supplement: Table S1 — Pathology of 39 Lymph Node Metastases. [file Table_1.DOCX]

**Table S1** Pathology of 39 Lymph Node Metastases

| Sourced cancerous foci | N | Classification |
| --- | --- | --- |
| hypopharynx | 2 | moderately differentiated squamous carcinoma |
| nasopharynx | 4 | poor-differentiated squamous carcinoma |
| parotiod gland | 2 | poor-differentiated mucoepidermoid carcinoma |
| salivary gland | 1 | poor-differentiated adenocarcinoma |
| tongue | 1 | Moderately and poor-differentiated adenocarcinoma,with mucinous adenocarcinoma) |
| thyroid | 5 | papillar carcinoma |
| lung | 5 | small cell cancer |
|  | 7 | poor-differentiated adenocarcinoma |
|  | 1 | small cell neuroendocrine carcinoma |
| breast | 5 | poor-differentiated adenocarcinoma |
| ovary | 2 | poor-differentiated adenocarcinoma |
| stomach | 1 | moderately and poor-differentiated adenocarcinoma |
|  | 1 | small cell neuroendocrine carcinoma |
| colon | 1 | poor-differentiated adenocarcinoma |
| unknown primary site | 1 | poor-differentiated adenocarcinoma |
|  | 1 | sarcomatoid carcinoma, |
